# Stroking in early mother-infant exchanges: The role of maternal tactile biography and interoceptive sensibility

**DOI:** 10.1371/journal.pone.0298733

**Published:** 2024-03-07

**Authors:** Isabella Lucia Chiara Mariani Wigley, Eleonora Mascheroni, Massimiliano Pastore, Sabrina Bonichini, Rosario Montirosso

**Affiliations:** 1 Department of Developmental and Social Psychology, University of Padua, Padua, Italy; 2 0-3 Center for the at-Risk Infant, Scientific Institute IRCCS E. Medea, Bosisio Parini, Italy; University of Modena and Reggio Emilia: Universita degli Studi di Modena e Reggio Emilia, ITALY

## Abstract

Caress-like is a crucial component of caregiving and a key factor in mother-infant interactions. Mother’s experience of touch during her own childhood (i.e., tactile biography) has been found to be related to maternal actual use of caress-like touch (i.e., stroking) during mother-infant exchanges. Evidence also suggests that maternal interoceptive sensibility (i.e., self-perceived sensitivity to inner-body sensations) might be related to sensitive caregiving abilities. However, further empirical investigation is needed to understand to what extent tactile biography and interoceptive sensibility have an impact on mothers’ stroking when interacting with their infants. Using an online survey, this cross-sectional study explored the potential association between maternal tactile biography, interoceptive sensibility and use of touch for interaction with their own infants in a group of 377 Italian mothers (mean age = 33.29; SD = 4.79). We tested and compared a series of multivariate linear mediation models using maternal tactile biography as predictor, maternal use of affective touch as outcome variable and Multidimensional Assessment of Interoceptive Awareness (MAIA) subscale scores as mediators. We found that, if a mother had positive touch experiences in her own childhood, she may be more likely to use touch in a positive and nurturing way with her own infant (i.e., stroking). Furthermore, mothers’ interoceptive sensibility in the form of attention regulation, self-regulation and body listening mediates the association between their past experiences of positive touch and their use of caress-like touch in mother-infant exchanges. This study highlights that maternal tactile biography is directly associated with mothers’ use of caress-like touch and indirectly linked to it through the mediating role of interoceptive sensibility.

## Introduction

Mother-infant exchanges are the first and most pervasive context in early life so that caregiver’s behavior critically contributes to define infants’ developmental outcomes in later life [1, 2]. This underscores the significance of caregiving sensibility, which refers to the aptitude of a caregiver to discern and respond to the emotional needs of the child, thus playing a crucial role in shaping developmental trajectories. Among other components of caregiving (e.g., affection, encouragement, responsiveness, intrusiveness), touch is known to be a central feature of the responsive and available caregiving environment and has a significant impact on infants’ neurobehavioral development and regulation [3–7]. Touch represents a powerful and nuanced mode of communication, facilitating the conveyance of emotional support, reassurance, and affection. Mothers can intentionally initiate a variety of tactile interactions with their children, serving various purposes [8]. These interactions encompass gentle and unhurried touches, resembling caresses indicative of affectionate touch (i.e., stroking). Alternatively, maternal touch can manifest as rapid tickling and lifting, creating playful stimuli (i.e., playful touch) or it may take on a sustained and prolonged nature, functioning to support the child’s regulation of behavior (i.e., holding touch). Additionally, maternal touch can be directed to maintain children’s attentional focus (i.e., attention-getting touch). In essence, distinct types of touch convey specific messages to the child and have been linked to specific bio-behavioral outcomes. For instance, the parasympatho-inhibitory regulation of infants aged 4–16 weeks increased while mothers were stroking them [9] and infants as young as 9 months demonstrate decreased heart rate and increased engagement in response to caress-like touch [10]. Furthermore, stroking regulates infants’ negative emotions and can assist in the modulation of their behavior towards people, object exploration and emotion processing [11–13]. Thus, to some extent, maternal touch reflects the quality of caregiving, so that lower maternal sensitivity results in lower exposure to maternal touch [14]. Importantly, touch is not just an infant-focused experience, it includes mutual involvement between caregiver and the infant. Therefore, one might wonder to what extent maternal tactile behaviors rely on the mother’s own feelings about touch. While the effects of caress-like touch on infant development have been widely investigated, subjective dimensions related to maternal sensitivity in the form of tactile behaviors did only recently begin to be considered [15]. However, further empirical investigation is needed to understand to what extent the mother’s positive experience of touch during her own childhood (i.e., tactile biography) has an impact on the current use of caress-like touch during interactions with her infant. Sensitive caregiving abilities could be also related to the sense of own internal bodily states (i.e., interoception) [16]. In fact, interoception was associated with sensitivity to others’ emotions and social emotions [17], suggesting that perception of one’s own body signals is crucially involved in the ability to understand emotion in others [18]. Moreover, interoceptive input triggered by a social interaction contributes to emotion-related effects of social touch [19]. In light of this, the overall aim of this study in a group of Italian mothers was to explore possible associations between self-perceived past affective touch experiences (i.e., tactile biography), interoception and use of caress-like touch toward their own infants. Identifying specific touch-related experiences associated with caregiving experiences is key to advancing conceptual models and develop innovative interventions when focusing on early mother-infant exchanges and caregiving environment.

### The role of positive past tactile experiences

Recent evidence suggests that life-long experiences of touch and levels of touch exposure may affect how individuals currently experience and perceive touch [20]. In the general population, experiences of positive tactile experiences during childhood and adolescence have been found to be associated with attachment styles and the use of stroking behaviors in adult age [21]. Specifically, avoidant attachment style seems to play as a mediator in the link between past (childhood/adolescent) and future (adult) experiences of caress-like touch. Individuals with avoidant attachment style tend to limit interpersonal touch in adulthood. Furthermore, Authors reported that avoidant attachment style is associated with the amount of stroking experienced in early life and the perception of touch deprivation later in life [21]. This implies that previous positive tactile experiences appear to be predictive of attachment, and in turn, attachment predicts current tactile behaviors highlighting, again, the key role of past positive tactile experiences. Studies focused on maternal tactile biography provided several insights [20, 22]. For instance, a mother’s feeling regarding her own history of touch interacts with her own use of touch in the relationship with her infant, which in turn is linked to the infant’s attachment style [23]. These findings indicate that the mother’s attachment security may be a core component of her internal working model of attachment which is transmitted to her child, potentially through some facets of her nurturing touch [24]. According to a study on young adults, lower self-reported frequencies of early parental touch during preschool age were predictive of higher depression and contribute to a poorer image of an individual’s romantic partner [22]. Similarly, using self-reports, a recent study documented that mothers’ self-perception of caress-like touch enacted in the relationship with their infants was linked to their childhood experiences of caress-like touch, to how mothers perceive touch in their own lives and to the amount of stroking experienced in their everyday life as adults [20]. The more positive touch-related experiences the mother had in her own childhood and the more comfortable she felt with caress-like touch in her every-day life, the more likely her appreciation and use of stroking during mother-infant exchanges. In sum, life-long caregivers’ experiences of touch seem to play an important role in predicting mothers’ use of touch during mother-infant interactions.

### Maternal interoception

It has recently been suggested that mothers’ awareness of her own internal states might support sensitive caregiving abilities [25]. Interoception, which is defined as the perception of physiological sensations within the body, is regarded as the foundation of subjective emotional states, and it is also highly associated with emotional identification and regulation [26, 27]. In line with this perspective, a recent qualitative study revealed that mothers’ sensitivity of internal states supports caregiving activities and provides insights into how mothers modulate their emotions in daily mother-infant interactions, included social touch exchanges [28]. Results show that mothers who perceived their body sensations as expressions of emotional experiences also recognized the reciprocal effect that body sensations could have on their own and their child’s emotional state. In contrast, bodily sensations were inaccessible to mothers who were more inclined to rely on formal knowledge (e.g., information from books, websites, etc.) during mother-infant exchanges instead of “listening” and “following” their own sensations based on their own and their infant’s body signals. Additionally, an association between a specific dimension of interoception, namely interoceptive sensibility, and social competences was suggested [29]. Interoceptive sensibility refers to the subjective tendency to perceive, appraise and use physiological signals, and it could be measured by self-report questionnaires such as the Multidimensional Assessment of Interoceptive Awareness (MAIA), which can capture judgments, beliefs, attitudes, thoughts, and feelings about the individual’s perception of interoceptive signals [30, 31]. For instance, interoceptive sensibility as expressed by MAIA scores was associated with sensitivity to others’ emotions [17], suggesting that the perception of one’s own body signals is crucially involved in the ability to understand emotion in others [18]. Consistently, mothers’ interoceptive sensibility predicted lower somatic problems through their sensitive caregiving behavior [32]. In other words, maternal interoceptive abilities were associated with more sensitive caregiving behaviors which, in turn, were associated with child developmental outcomes. In addition, mothers’ interoceptive knowledge about their own emotions was linked to social affective skills in their children (i.e., emotion regulation, social initiative, cooperation, self-control) in middle childhood [33]. Again, a higher level of maternal interoceptive sensibility predicts optimal later-life outcomes in 8-year-old children. Overall, this suggests that mothers’ ability to perceive their own bodily sensations influences their ability to interpret and empathize with their children’s emotional states through body signals. In light of this, one could wonder if maternal interoceptive sensibility is not only related to sensitive caregiving but also to maternal caress-like touch behaviors which is a key component of sensitive caregiving.

### The present study

Our study was aimed at exploring possible associations between maternal tactile biography, interoceptive sensibility and use of caress-like touch in mother-infant exchanges. We operationalized: 1) maternal self-perception of tactile biography using the Childhood/Adolescent Touch Experience scale of the Tactile Biography Questionnaire (TBQ) [21]; 2) touch behaviors during daily caregiving activities by the Stroking scores of the Parent-Infant Caregiver Touch Scale (PICTS) [34]; 3) interoceptive sensibility using the MAIA subscale scores [35]. Using a series of linear multivariate mediation models, we investigated if maternal tactile biography was predictive of maternal use of stroking during mother-infant interactions (direct effect) and if interoceptive sensibility (indirect effect) played a mediating role in use of caress-like touch. We expected that self-perception of mothers’ use of stroking in caregiving activities could be, at least partially, explained by maternal tactile biography. We also hypothesized that maternal interoceptive sensibility would play a role in this association. Specifically, we expected that mothers who reported more touch experience throughout childhood would show increased interoceptive sensibility and, in turn, more caress-like tactile behaviors with their infants.

## Materials and methods

### Procedure and participants

An online anonymous survey set up with Qualtrics was conducted between May and August 2021. Mothers’ inclusion criteria were: (a) age ≥18 years, (b) having a full-term infant (born with a gestational age ≥37 week) in the past 18 months, (c) being Italian native speakers, (d) reporting no self-perceived depressive, anxiety and stress symptoms. Participants were recruited using snowball sampling, and they provided informed written consent prior to the survey. The survey was completed by 562 mothers. Sociodemographic information included general information such as age, civil status, educational level, gender, age, birth weight, and gestational age. As the data collection was performed during the COVID-19 pandemic, a specific section on the survey was used to collect information about the impact of the COVID-19 situation on participants (see S1 Fig in S1 File). Participants with missing data accounting for more than 25% of the survey were excluded from further analysis. Respondents with 85% completion rate who did not however fulfill eligibility criteria were excluded from the final sample. Included and excluded participants were compared for infants’ age, gender, birth weight, gestational age, and maternal age, and no differences were found (S1 Fig in S1 File). The final sample included 377 mothers, and their complete descriptive statistics are provided in Table 1. Our sample size was more than acceptable given that the N:p ratio—where N is the number of participants and p is the number of indicator variables—was above 10 [36]. The entire procedure was reviewed and approved by the University of Padua ethics committee (protocol number: 6726ADCF521BE52EDBF4DC6C4A0485B9). Recruiting and testing were in line with the local Ethics Committee requirements and the Declaration of Helsinki.

**Table 1:** Descriptives of the included subjects.

	n	mean	sd	min	max	skew	kurtosis
Infant age	376	9.73	5.28	1.00	19.00	0.26	-1.05
Mother age	377	33.29	4.76	19.00	47.00	0.10	-0.11
Birth weight	377	3361.37	429.52	958.00	4595.00	-0.26	1.88
Gestational age	377	39.84	1.24	37.00	42.00	-0.24	-0.55

Civil state				
	Divorced/Separated	Married/Cohabitant	Single	Widower
n	23	331	9	1

Study title				
	Middle school	High school	Bachelor degree	Master degree/PhD
n	17	123	176	56

### Measures

#### Self-perceived depressive, anxiety and stress symptomatology

Levels of depression, anxiety and stress as mental health markers were measured using the Italian version of the Depression, Anxiety and Stress Scale—21 Items (DASS-21) [37]. The DASS-21 [38] consists of three self-report scales, each with 7 items rated on a 4-point Likert scale. Participants are asked to indicate to what extent a given statement applied to them over the past week (ranging from 1 “Does not apply” to 5 “Applies very much”). Scores for depression, anxiety and stress are calculated by summing the relevant item scores. For each DASS scale, cut-off scores were developed to define mild/moderate/severe/extremely severe scores. Recommended cut-off points are reported in S1 Table in S1 File McDonald’s Omegas were .90, .88, .83 for depression, anxiety and stress respectively.

#### Maternal tactile biography

Maternal tactile biography was assessed using the Italian version of the TBQ [39]. The TBQ [21] is made of 28 items rated on a 5-point Likert scale (ranging from 1 Never to 5 Very frequently) grouped into 4 components namely; Childhood/Adolescent Touch Experience, Comfort with Interpersonal Touch; Fondness for Interpersonal Touch, Adult Touch Experience. Only the first component (Childhood/Adolescent Touch Experience) was considered in this study as we wanted to evaluate mothers’ experience of touch during their childhood and adolescence. This scale measures the extent to which mothers had positive experiences of affective touch and body contact in close relationships during their childhood and adolescence. McDonald’s Omegas was.89.

#### Maternal stroking

Mothers’ perception of their experiences of touch toward their infants was measured using the Italian version of the PICTS [20]. The PICTS [34] is a 12-item parent report evaluating how often a mother stroked her infant’s back, head, tummy, arms, legs and how often she picked him/her up or cuddled, rocked, kissed, and held him/her. The PICTS items can be grouped into three different factors called Stroking, Affective Communication and Holding. The Stroking factor relates to caregiver caress-like touch behaviors and includes items such as: “I stroke my baby’s tummy” and “I stroke my baby’s leg”. The Affective Communication factor is focused on caregiver behaviors aimed at demonstrating affection to the infant in routine and face-to-face interactions such as talking, kissing and watching (e.g., “I kiss my baby”, “I talk to my baby”). The Holding factor refers to holding and other behaviors that involve close physical contact between caregiver and infant (e.g., “I rock my baby”, “I hold my baby”). Mothers were requested to indicate the frequency of their engagement in those behaviors using a 5-point Likert scale that ranges from Never (1) to A Lot (5), with higher scores indicating a higher frequency of stroking behaviors. The PICTS showed good psychometric properties [34] and it has already been used in studies with Italian participants [40]. For the aim of the present study, only the Stroking scale was selected and its McDonald’s Omega was.81.

#### Mother’s interoceptive awareness (awareness of mind-body integration)

The Italian version of the Multidimensional Assessment of Interoceptive Awareness (MAIA) [41] was employed to measure mother’s interoceptive sensibility. The MAIA questionnaire [35] consists of 32 items evaluating eight aspects of interoception (i.e., Noticing, Not-Distracting, Not-Worrying, Attention Regulation, Emotional Awareness, Self-Regulation, Body Listening, Trusting). Respondents are asked to indicate how often each statement applies to them generally in daily life on a 5-point Likert scale ranging from Never (1) to A Lot (5), with higher scores indicating a higher frequency. Concerning specific subscales, the Noticing scale refers to an individual’s ability to be aware of body sensations, including comfortable and uncomfortable ones. This scale counts items such as “I notice changes in my breathing, for example if it slows down or speeds up”. The Not-Distracting and Not-Worrying subscales contain items evaluating a tendency to ignore or become stressed because of sensations of pain or discomfort. The Attention regulation scale refers to the individual’s ability to sustain and control attention to body sensation. Emotional awareness includes items that address the recognition of the link between bodily sensations and emotional states (e.g., I notice how my body changes when I am angry). The Self-regulation subscale refers to the ability to regulate psychological distress by focusing attention on body sensations (e.g., I can use my breath to reduce tension) and the Body listening subscale assesses one’s ability to focus on bodily sensations for psychological insight (e.g., I listen to my body to help me choose what to do). Finally, the Trusting subscale includes items related to individual’s experiences of body as safe and trustworthy. We computed McDonald’s Omegas for all scales: Omega values for Not-Worrying, Attention Regulation, Emotional Awareness, Self-regulation, Body listening and Trusting subscales were .83, .87, .81, .80, .85 and .90, respectively. McDonald’s Omegas for Noticing and Not-Distracting subscales were extremely low (.65 and.20) indicating unacceptable performances on these scales. For this reason, these scales were excluded from subsequent analysis.

### Analysis plan

Descriptive statistics and bivariate associations between the included variables (TBQ- Childhood/adolescent touch experience score, MAIA-subscales scores, and PICTS- Stroking scale) were presented. In order to interpret results, we considered an effect size of Pearson’s r as low if r was around.10 or less, as medium if r was around.30, and large if r was higher than.50 [42]. Internal consistency was evaluated through McDonald’s Omegas, and scales with low McDonald’s Omega values were excluded from subsequent analysis as data were judged unreliable. Afterwards, we ran a series of linear multivariate mediation models including maternal tactile biography as predictor, maternal stroking as outcome and MAIA subscales as mediators. One model was set up for each MAIA subscale allowing to test one mediator at a time. In order to evaluate tested models, a model comparison approach was used as it allows to determinate whether mediation terms improved the predictive power of the model. Mediation models were compared with an overall mediation model (including all the mediators), a null model and a main effect model (including only the predictor) using the Akaike information criterion (AIC) [43] and Akaike weights [44]. According to AIC criteria, the lower the value, the better the predictive power of the model. Akaike weights range from 0 to 1, the higher the value, the better the model is at describing data accurately [43, 45]. Model comparison based on AIC [43] and AIC weights [44] also enabled us to explore which of the possible mediators had the greatest weight in explaining the link between maternal tactile biography (i.e., predictor) and maternal caress-like touch behaviors (i.e., outcome). For a detailed description of this second step, model comparison approach and information criteria, see the (section 3 page 9 in S1 File). It should be noted that, while some studies have highlighted that maternal touch behaviors may vary based on the infants’ age and gender [46, 47], our previous findings within the same sample demonstrated no differences in maternal touch behaviors among infants aged 3, 6, 12, and 18 months, as well as across genders [20]. Consequently, we did not include these variables in our models. However, recognizing recent research indicating potential variations in interoceptive awareness based on age [48], we have incorporated maternal age as a covariate for model fitting. We invite the reader to refer to the supplementary material of our previous work for further details [20]. All the analyses were run using statistical software R [49] and the lavaan package for model estimation [50].

## Results

### Descriptive statistics

Sociodemographic characteristics of participating mothers and infants and maternal mental health data are summarized in Table 1. Depressive symptoms, anxiety, and stress scores were within the lower cut-off of DASS-21, so participating mother were at low mental health risk. Descriptive statistics regarding maternal tactile biography (TBQ), interoceptive sensibility (MAIA) and Stroking scale (PICTS) and bivariate correlations between these variables are summarized in Table 2. The percentage of missing data was low (S3 Fig in S1 File). As a result, a listwise deletion strategy was performed excluding subjects with missing values from the analyses.

**Table 2:** Descriptives and bivariate correlation coefficients of the included variables (n = 377).

	mean	sd	1	2	3	4	5	6	7	8
1.Not_worrying	3.51	0.89	1							
2.Attention_regulation	3.61	0.99	0.01	1						
3.Emotional_awareness	4.44	1.06	0.16	0.19	1					
4.Self_regulation	3.49	1.14	0.08	0.25	0.4	1				
5.Body_listening	3.34	1.13	0.16	0.13	0.57	0.45	1			
6.Trusting	4.14	1.21	0.18	0.2	0.58	0.5	0.7	1		
7.Maternal_stroking	3.71	0.70	0.33	0.12	0.46	0.32	0.55	0.55	1	
8.Tactile_biography	3.54	0.81	0.25	0.05	0.24	0.26	0.26	0.25	0.17	1

### Multivariate linear mediation models

**Fig 1:**
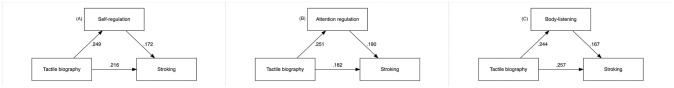
Multivariate linear mediation models (n = 342). Maternal past and positive tactile experiences (i.e., TBQ-Childhood/Adolescent Touch Experience) are linked to actual stroking behaviors (i.e., PICTS-stroking) directly and through interoceptive sensibility in the form of Self-regulation (A), Attention regulation (B) and Body listening (C) (i.e., MAIA-subscales). Standardized coefficients are reported.

All the tested models have maternal tactile biography (TBQ; Childhood/Adolescent Touch Experience subscale) as predictive factor, maternal interoceptive sensibility (MAIA Trusting, Not-Worrying, Attention regulation, Self-regulation, Emotional awareness and Body listening subscales) as candidate mediators, and maternal caress-like touch as outcome (PICTS; Stroking subscale). Overall, as it clearly emerges from AIC values, mediation models with one mediator at a time outperformed the overall, the null and the main-effect models (Table 3). Among the MAIA subscales tested as mediators, Attention regulation, Self-regulation and Body listening were significant mediators in the association between maternal tactile biography and maternal use of stroking in the relationship with her infant. Standardized estimates of these models are reported in Fig 1 and the parameters for indirect and total effects as well as the proportion of variance explained for each outcome variable (i.e., R-square) are reported in Table 4. The indirect effect represents the influence of the independent variable on the dependent variable through the mediator(s). It is calculated as the product of the path coefficients linking the independent variable to the mediator and the mediator to the dependent variable. The total effect represents the overall impact of the independent variable on the dependent variable, considering both direct and indirect pathways. It is the sum of the direct effect, the pathway from the independent variable directly to the dependent variable without going through any mediator, and the total indirect effects through all mediators. Trusting, Not-Worrying and Emotional awareness were non-significant mediators and coefficients of the respective models are reported in section 3 of the Supplementary Material. Section 3.2 of the Supplementary Material summarizes the results of the mediator comparison using the AIC weights which will not be discussed below.

**Table 3:** AIC and AIC weights. Each row represents a model named after the mediator (i.e., a subscale of the MAIA). The overall model incorporates all the mediators together, the main effect model considers only predictor and outcome, while the null model assumes no relationships between the variables.

	df	AIC	weights
Not_worrying	8	1773.84	≈ 1
Attention_regulation	8	1840.11	<.001
Emotional_awareness	8	1859.34	<.001
Body_listening	8	1924.04	<.001
Self-regulation	8	1926.36	<.001
Trusting	8	1949.58	<.001
Overall	28	6900.35	<.001
Main_effect	16	6976.29	<.001
Null	18	9844.24	<.001

**Table 4:** Indirect and total effects of maternal past and positive tactile experiences (i.e., TBQ-Childhood/Adolescent Touch Experience) on actual stroking behaviors (i.e., PICTS-stroking) through Self-regulation, Attention regulation and Body listening (i.e., MAIA subscales).

	Mediators								
	Self-regulation			Attention regulation			Body listening		
	*β*	p	$R^{2}$	*β*	p	$R^{2}$	*β*	p	$R^{2}$
			0.106			0.106			0.106
Indirect effect	0.037	0.019		0.035	0.024		0.043	0.009	
Total effect	0.286	<0.001		0.286	<0.001		0.287	<0.001	

## Discussion

Along with other components of sensitive caregiving, caress-like touch is considered to be a crucial factor in early caregiver-infant exchanges [3]. As most studies have so far investigated stroking correlates in infants, we shifted the focus from infant to mother exploring possible subjective dimensions linked to her self-perception of caress-like tactile behaviors. Specifically, we investigated if maternal tactile biography was predictive of maternal use of stroking during mother-infant interactions (i.e., direct effect) and if interoceptive sensibility (i.e., indirect effect) had a mediating role on use of caress-like touch. Our findings suggest that mothers’ past positive touch experiences influence their use of stroking supporting the first hypothesis, namely mothers experiencing a greater positive touch experience throughout childhood showed an increased use of caress-like touch with their infants. This is in line with previous studies suggesting that mother’s feelings regarding her own history of touch interacts with her current use of touch in mother-infant exchanges, which in turn might influence the infant’s attachment security [23], [39]). We hypothesize that mothers with high exposure to touch in their own childhood may perceive touch as an important channel for interpersonal affective exchanges. In this respect, a recent study on a non-parent sample documented that the degree of exposure to tactile experience shapes the perception of caress-like touch [51]. In particular, individuals who had minimal exposure to touch in daily social interactions demonstrated a decreased ability to distinguish between various types of stroking velocities and rated caress-like touch as less pleasant, control participants reported enjoying touch from close individuals. This suggests that, the greater the experience of interpersonal touch, the more its impact on our perception of stroking and on its use. For this reason, maternal touch biography may be a key factor when examining touch in mother-infant relationships. As an indirect effect, our results show that mothers’ interoceptive sensitivity plays a significant role in the association between their past positive touch experience and their current use of touch. This highlights two interesting associations: 1. One between tactile biography and interoceptive sensibility; and 2. One between interoceptive sensibility and maternal caress-like touch behaviors. First, mothers exposed to more positive touch throughout their childhood reported a higher level of interoceptive sensibility. This corroborates previous evidence suggesting that early social experiences have an impact on the development of interoceptive sensibility [52]. According to a recent retrospective study, young adults categorized as exhibiting indications of avoidant attachment displayed reduced levels of interoceptive awareness [53]. This means that, insensitive caregiving –which underlies avoidant attachment style– may impair a child’s ability to form accurate representations of bodily sensations [53, 54]. It should be noted that tactile biography predicted specific aspects of interoceptive sensibility such as self-regulation, body listening and attention regulation. Thus, a mother’s past positive touch experience does not affect her current inner body perception in a general way but rather in terms of her ability to: 1. regulate psychological distress by focusing attention on body sensations, 2. focus on bodily sensations for psychological insights, 3. sustain and control attention to body sensation. How can we interpret these results? Tactile parent-infant interactions and skin-mediated signals, including caress-like touch, has been recently redefined as interoceptive stimuli [15]. Furthermore, it has been suggested that touch experiences in childhood might promote interoception, which in turn has been related to bodily self-perception [52]. The sense of touch and skin-to-skin contact, primarily through the allostatic function of the mother which continuously informs about the physiological condition of the infant’s entire body, can promote the capacity to use interoceptive signals to detect bodily self [55]. Thus, we speculate that mothers with a greater touch experience developed greater integration of interoceptive and exteroceptive information, an essential process for the individual’s homeostasis and for the development of bodily self which, in turn, supports the subjective experience (first-person perspective) of inner body perception [52]. Second, a higher level of interoceptive sensibility was associated with more stroking behaviors during mother-infant exchanges. Specifically, self-regulation, body listening and attention regulation predicted mothers’ use of caress-like touch in interactions with their infants. Our results are in line with the idea that increased maternal embodied feelings and body sensations might support social interactions, also promoting the use of caress-like touch as a key communicative channel. For instance, the speed of intuitive maternal stroking in infants ranging from 4 to 54 weeks old exhibited a significant correlation with maternal interoception, as assessed through heart rate measurements [56]. This implies that certain aspects of maternal interoception may modulate maternal caress-like touch behaviors. Accordingly, the present study shows that maternal interoceptive sensibility is crucial, not only for processing mothers’ emotional experience and self-regulation [57] but also in supporting caress-like and stimulating touch towards their infants. More broadly, mothers’ ability to identify signals from their own body might support their emotional experience which, in turn, can modulate mother-infant exchanges [58]. The interoceptive knowledge of mothers regarding their own emotions has been linked to children’s social affective skills, encompassing aspects such as emotion regulation, social initiative, cooperation, and self-control [33]. Thus, one can speculate that mothers with heightened interoceptive sensibility may be more attuned to their own bodily sensations and emotions which in turn could help them to be more responsive to their infants’ needs using touch. However, while our study suggests a potential link between interoceptive sensibility and the frequency of touch in parent-infant interactions, it is crucial to note that our use of a self-report measure only captures touch frequency and does not provide comprehensive information about maternal responsiveness. In parent-infant interactions, considering not just the amount but also the context and appropriateness of touch is of paramount importance [3]. In light of these considerations, our results might provide insights for parental intervention programs. Considering the cascade effects that maternal tactile biography seems to have on key components of sensitive parenting (i.e., maternal self-perceived interoception and caress-like touch behaviors), exploring mothers’ past positive touch experiences could be a favorable starting point to explore maternal embodied dimensions. In this regard, maternal interoceptive sensitivity could be targeted in support interventions for mothers with typical and atypical infant development. In typical infant development, a recent qualitative study suggests that supporting mothers’ attention to their own emotions and body sensation modulates infant care and how mothers regulate emotions in the post-natal period [28]. Similarly, mothers of infants with neurodevelopmental disabilities showing awareness of their own bodily signals considered their children’s bodily signals as significant and used them to regulate interaction [25]. This suggests that supporting mothers to “tune in” to their own internal states and encouraging them to use this awareness in their exchanges with their infants might be key to improving both their mental well-being as well as their ability to detect and respond to their infant’s signals. Moreover, while disrupted interoception was associated with several mental health conditions such as anxiety, mood, eating, addictive and somatic symptom disorders [59–61], no study has so far explored this association in caregivers –not even during sensitive periods such as post-partum. If we could better identify specific mechanisms associated with interoceptive sensibility and caress-like touch, we could design more effective interventions to support parenthood in typical and atypical situations. The Video Feedback Intervention (VFI), for instance, is an early family-centered care approach that has proven effective in promoting responsive parenting and enhancing infants’ behavioral and socioemotional development in typical and atypical conditions [62, 63]. Within this intervention framework, as previous works suggested, a focus on maternal bodily sensation, interoceptive sensibility and observed caress-like touch is both feasible and potentially highly impactful [25, 64]. The present study has some limitations which are important to mention. First, our participants were recruited by convenience sampling—a method which may prevent generalization of findings. Second, this study lacked “objective” measurements of maternal interoception and caress-like touch behaviors as well as measurement of contextual aspects of these dimensions. Integrating future research with biomarkers of interoceptive abilities, such as heart-beat detection, could help us consider other facets of interoception (e.g., interoceptive accuracy) and thus open up new horizons for this field of research. Similarly, research methods such as video-taped mother-infant interactions could enable direct observation caregiver-infant exchanges and thus help code tactile behaviors directly through observational coding scales [8, 65, 66]. This study only used self-report scales, which could be influenced by so-called “faking good” responses. Even if they have already been widely used and have shown good psychometric properties suggesting that they map onto real-life behaviors and predispositions [23, 67–69], integrating an experimental design with more “objective” measurements would further improve the strength of data. Another constraint pertaining specifically to the evaluation of maternal tactile history is our reliance on mothers to recollect tactile experiences from childhood and adolescence. The ambiguity arises as to whether these mothers genuinely lived through these experiences or if they retrospectively reinterpreted certain aspects. An additional improvement consists in the inclusion of infants’ behavioral and affective outcomes in order to explore possible associations between infant and maternal dimensions. Lastly, our study participants were mothers at low risk for mental health. This limits generalization of our findings to high-risk mothers. Evidence suggests that frequency and quality of early touch during mother-infant interactions are associated with maternal mental health. Research highlighted that depressed mothers touch their infants less often and engage in less stroking during caregiving [70]. Previous evidence also suggests a link between body sensations and maternal mental health in term of depressive symptoms, anxiety, and stress levels [71, 72]. Future research could explore whether direct and indirect (via interoception) effects of maternal tactile biography on the use of caress-like touch during interactions are associated with depressive symptoms, anxiety and stress levels [25]. In conclusion, our findings provide evidence that mothers’ past positive touch experiences, interoceptive sensitivity and current use of caress-like touch are interconnected. Stroking behaviors may be influenced by maternal tactile biography and sensitivity of internal states. Gaining insights into how these subjective dimensions impact the present use of caress-like touch can provide valuable information for designing early intervention programs that aim to enhance nurturing parent-infant interactions in both typical and atypical development. Ultimately, this can contribute to fostering optimal outcomes in children throughout their lives.

## Supporting information

S1 File (PDF)
