# Supplementary material for: Stroking in early mother-infant exchanges: The role of maternal tactile biography and interoceptive sensibility
Source: PLoS One. 2024 Mar 7;19(3):e0298733. doi: 10.1371/journal.pone.0298733 (PMC10919687; doi:10.1371/journal.pone.0298733)
Supplement: S1 File — (PDF) [file pone.0298733.s001.pdf]

Supplementary Material for:

Stroking in early mother-infant exchanges: the role of maternal tactile biography and interoceptive sensibility

Isabella L.C. Mariani Wigley, Eleonora Mascheroni, Massimiliano Pastore, Sabrina Bonichini, Rosario Montirosso

Supplementary material edited by:

Isabella L.C Mariani Wigley* & Massimiliano Pastore

Department of Developmental and Social Psychology

University of Padua, Padua

e-mail address

**isabella.marianiwigley@unipd.it*

Contents

1 Introduction **3**

2 Descriptives **4**

2.1 Comparing original and current sample 4

2.2 DASS-21 cutt-off 5

2.3 Relative frequencies distribution of questionnaires scores 6

2.4 Percentage of missing values of PICTS, TBQ and MAIA items 7

3 Model Comparison **9**

3.1 Multivariate Linear Mediation Models 10

3.1.1 Overall Model 10

3.1.2 Not worrying model 14

3.1.3 Attention regulation model 15

3.1.4 Emotional awareness model 16

3.1.5 Self regulation model 18

3.1.6 Body listening model 19

3.1.7 Trusting model 21

3.1.8 Null model 22

3.1.9 Main effect model 24

3.2 Final Comparison 27

4 References **28**

1 Introduction

This report resumed supplementary information for the manuscript titled "Maternal interoceptive awareness bridges the association between maternal tactile biography and her present caress-like touch behaviors". The overall aim of the present study was to explore the role played by interoception in the association between maternal tactile biography and maternal stroking during daily caregiver activities. In this regard, caress-like touch is known to represent a crucial component of caregiving in early life and constitutes a key factor with a significant impact on infant later-life outcomes. Recent findings also suggest that maternal interoceptive awareness, which is individual ability to perceive signals coming from the own body, may represent an important factor in supporting the quality of care. As maternal tactile biography has already been found related to maternal present use of stroking during mother-infant exchanges, the overall aim of the present study was to explore a possible role played by maternal interoception. In order to test this association,data analyses were run on a sample of 377 Italian mothers (mean age = 33.29; SD = 4.79) participating in an online survey. We tested and compared a series of multivariate mediation models setting maternal tactile biography as predictor, maternal actual use of caress-like as outcome and MAIA-subscales as mediators. Findings suggest that mothers' interoceptive awareness, in form of attention regulation, self-regulation and body listening, mediates the association between maternal past experiences of positive touch during childhood and present caress-like touch enacted in mother-infant exchanges.

Here, we will focus only on the statistical analysis. For theoretical aspects, study aims,experiment design, and results interpretation the reader can refer directly to the article. Analysis were conducted with the R software version 3.6.1 (R Core Team, 2018) and models were estimated using the R package lavaan (Rosseel, 2017).

2 Descriptives

2.1 Comparing original and current sample

Participants inclusion criteria a) be older than 18 years old and b) having had a full-term baby in the past 19 months, c) be an Italian native speaker. The survey was filled by 562 mothers. Participants with data missing from more than 25% of items were eliminated from subsequent analyses. Similarly, participants who agreed to participate and completed the 85% of items but did not fulfill the eligibility criteria were excluded from the final sample. From the initial sample, n= 572 subjects were excluded. In order to explore if excluded and included mothers were comparable, we graphically explored the frequencies of gender, answers to yes/no questions about COVID-19 and the density of age and fear of COVID-19 (from 0 to 10) in the two groups figure S1. The two groups resulted comparable.

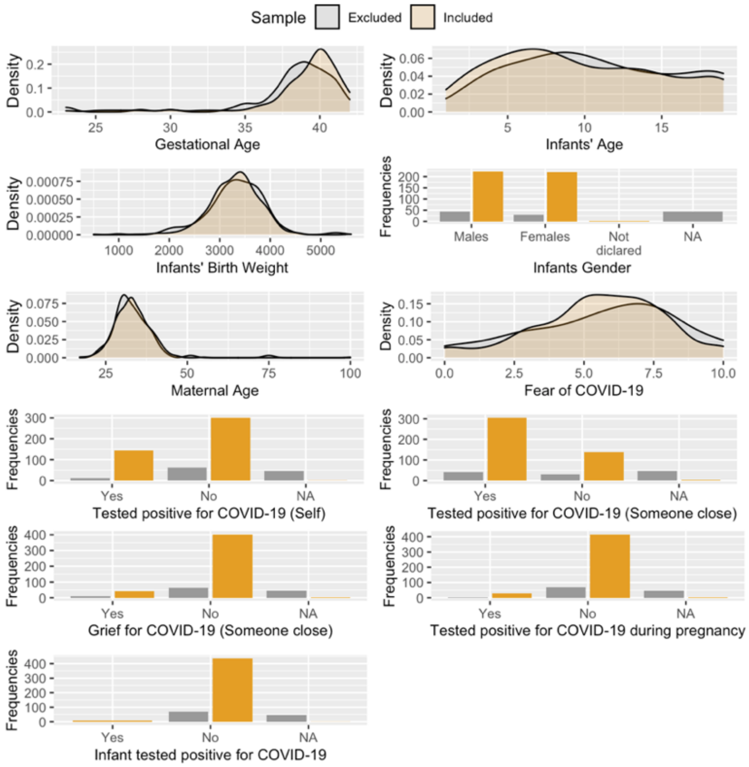

Figure S1: Comparing samples. Included label refers to participants who met the inclusion criteria. Excluded label refers to participants who did not met one ore more inclusion criteria.

2.2 DASS-21 cutt-off

The Depression, Anxiety and Stress Scale – 21 Items (DASS-21; Bottesi et al., 2015) is a self-report scale developed to assess the levels of depression, anxiety and stress. The DASS-21 (Lovibond & Lovibond, 1995) consists of three self-report scales, each with 7 items rated on a 4-point Likert scale. Participants are asked to indicate to what extent a given statement applied to them over the past week (ranging from 1 "Does not apply" to 5 "Applies very much"). For each DASS scale, cut-off scores were developed to define mild/moderate/severe/extremely severe scores. Recommended cut-off points are reported by Bottesi et al., 2015 and resumed here in table S1.

Category	Depression	Anxiety	Stress
Normal	0-9	0-7	0-14
Mild	10-13	8-9	15-18
Moderate	14-20	10-14	19-25
Severe	21-27	15-19	26-33
Extremely severe	28+	20+	34+

Table S1: DASS-21 cut-off scores.

2.3 Relative frequencies distribution of questionnaires scores

Figure S2 represent relative frequencies distribution of items scores of the included instruments. Items' response values and frequencies are reported on the x and y axis respectively.

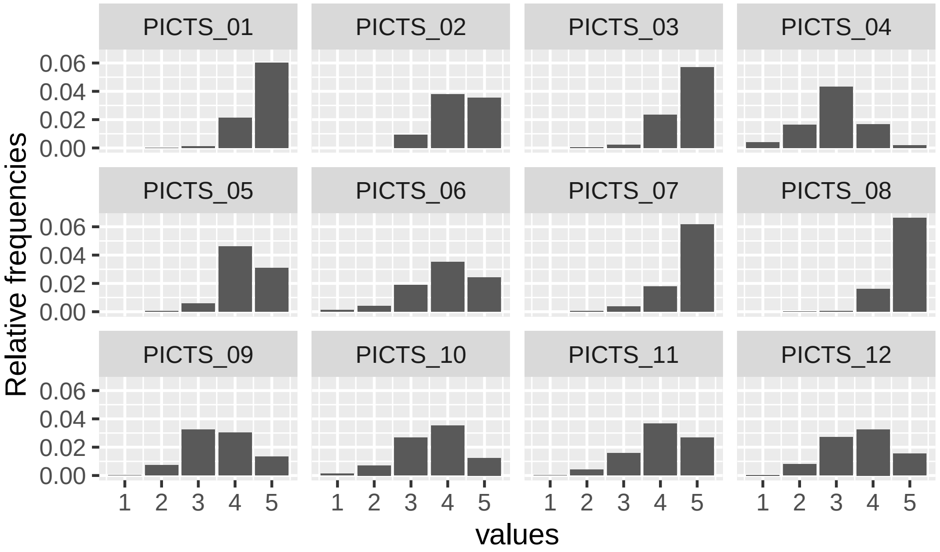

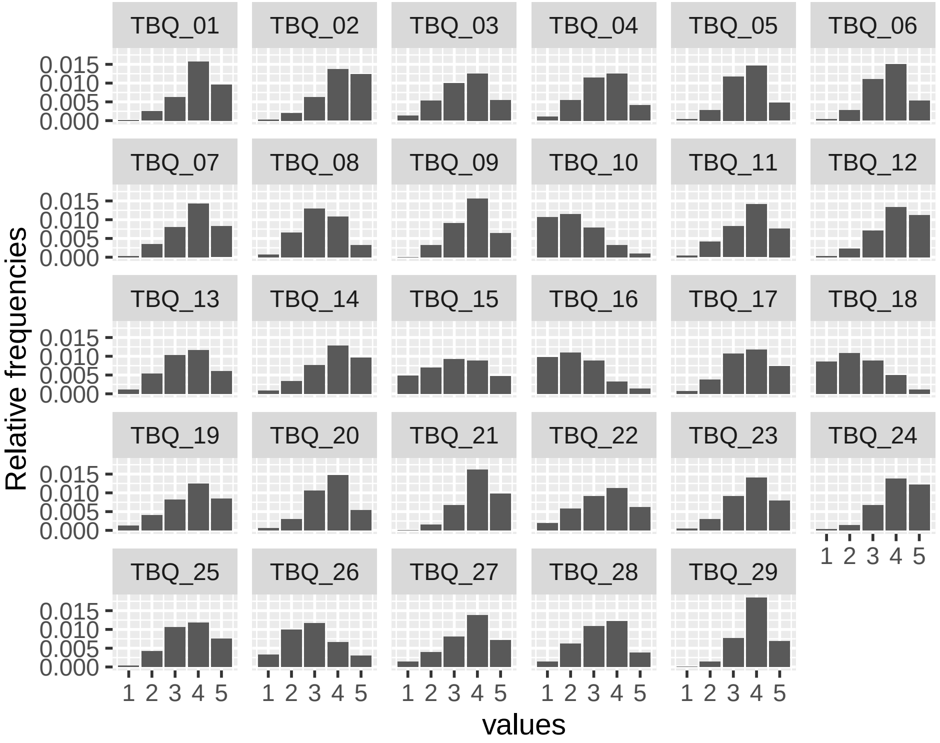

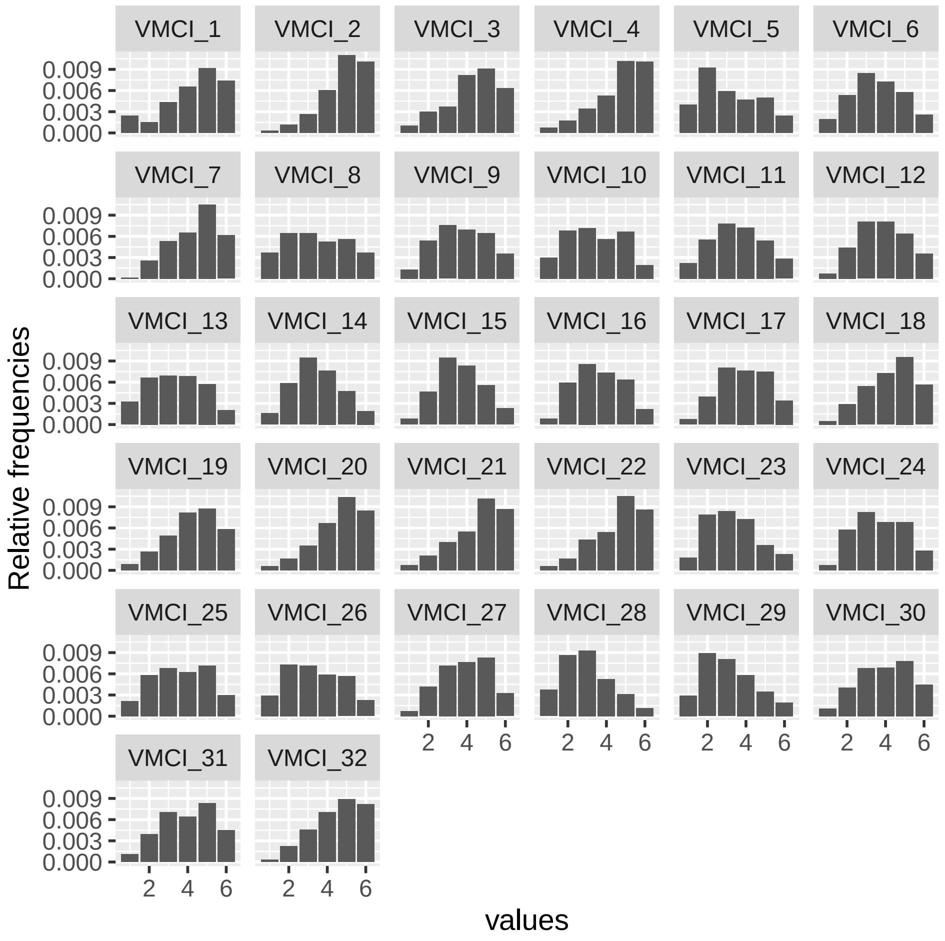

Figure S2: frequencies distribution of PICTS, TBQ and MAIA (n = 377). MAIA items are named here as VMCI.

2.4 Percentage of missing values of PICTS, TBQ and MAIA items

Fig. 3 represents the frequency (and percentage) of missing values for each item of PICTS, TBQ and MAIA questionnaires. Given the low percentage of missing data, a listwise deletion strategy was performed, excluding subjects with missing values from the analyses.

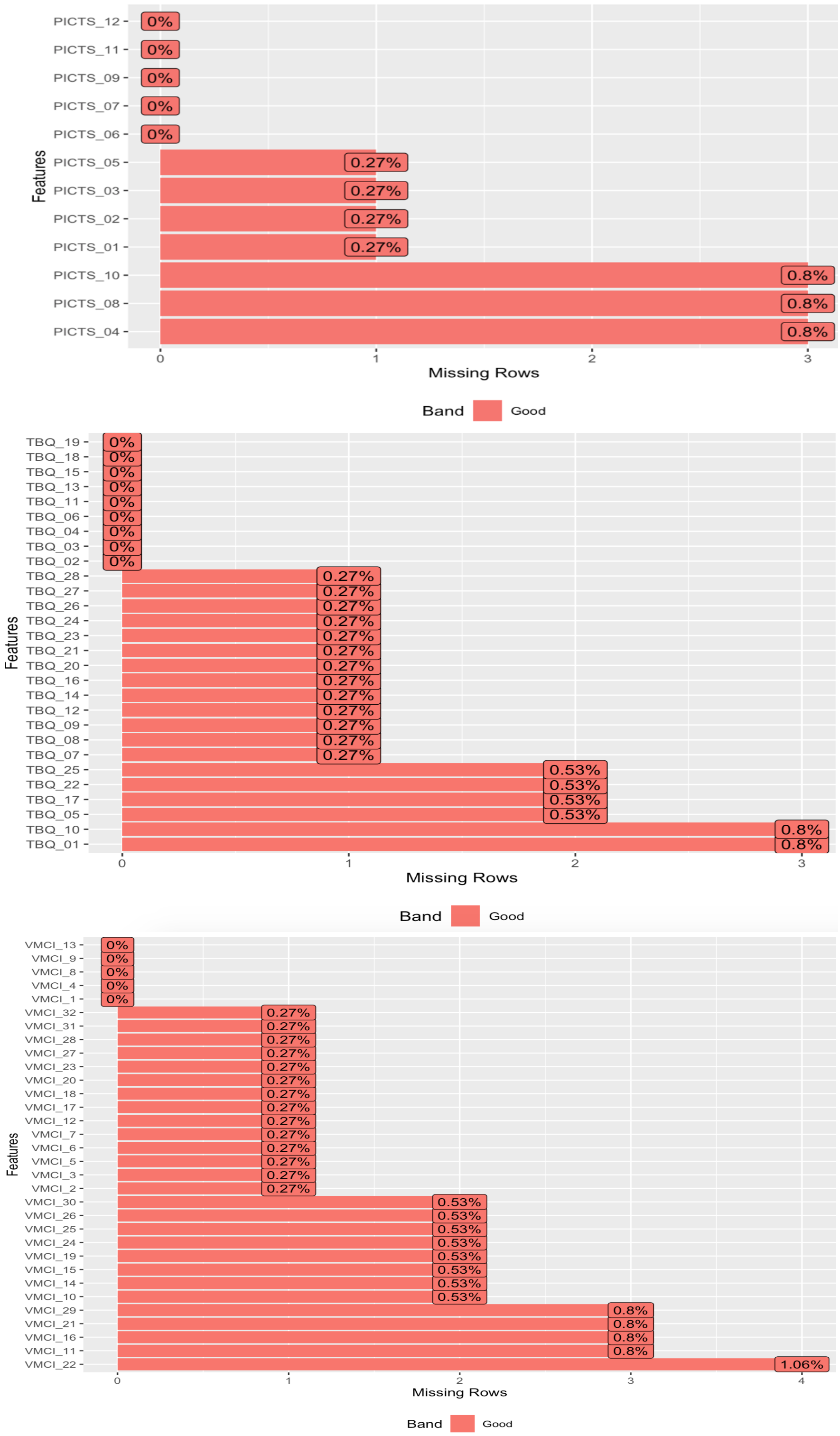

Figure S3: Percentage of missing values for each item of PICTS, TBQ and MAIA questionnaires (n = 377). Every row represents an item of PICTS, TBQ and MAIA respectively. MAIA items are named here as VMCI.

3 Model Comparison

In order to evaluate the research hypotheses about the role played by maternal interoception in the association between maternal tactile biography (i.e., TBQ-childhood and adolescents scores) and maternal actual use of caress-like touch in daily caregiver activities, a model comparison approach was used. Model comparison allows for the selection of the most plausible models given the data and a set of candidate models (McElreath, 2016). Models are compared in terms of statistical evidence (i.e. support by the data) using information criteria (Wagenmakers & Farrell, 2004), which enables the evaluation of models considering the trade-off between parsimony and goodness-of-fit (Vandekerckhove et al., 2015). In this regard, it is important to keep in mind that as the complexity of the model increases (i.e. more parameters) the fit to the data increases as well but generalizability (i.e. ability to predict new data) decreases (Pitt, Myung & Zhang, 2002). The researchers' aim is to find the best balance between fit and generalizability in order to describe, with a statistical model, the important feature of the studied phenomenon, but not the random noise of the observed data. Information criteria provide an estimate of the average deviance (i.e. error) of the model's ability to predict new data, thus lower values are interpreted as indications of a better model (McElreath, 2016). Given the structure of the data, a series of multivariate mediation models were set up and compared with with a null model and a main effect model (i.e., including only the predictor). In the light of what we said above, we used the AIC (Akaike, 1974) and Akaike weights (Anderson & Burnham, 2002) to compare tested models. According to AIC criteria, the lower the value the better the model is at predicting new data, while for Akaike weights, which range from 0 to 1, the higher the value, the better the model is at describing data accurately (Vandekerckhove et al., 2015; Wagenmakers & Farrell, 2004). Moreover, based on AIC (Akaike, 1974) and AIC weights (Anderson & Burnham, 2002), we also explored which of the possible mediators had the greatest weight in explaining the link between maternal tactile biography (i.e., predictor) and maternal present stroking (i.e., outcome). All the models are controlled for maternal age. We remind the reader that

Noticing and Not disturbing sub-scales were excluded from this comparison as omega values resulted .56 and .20 respectively, highlighting unacceptable performances of the scales.

3.1 Multivariate Linear Mediation Models

In this section we first specify all the multivariate mediation models tested. Subsequently we give the results of the model comparison. In order to read the following boxes, please note that parameters such as a, b1, d1, b2, d2, and so on, represent labels of coefficients in the regression equations. Each of these coefficients has a specific interpretation in the context of the model. For instance, *a* represents the regression weight linking the predictor (i.e., TBQ_child) to the latent variable (i.e., f1_picts). The *b* coefficients capture the direct paths between past positive experiences and present maternal tactile behaviors, while the *d* coefficients represent the effects of maternal past positive tactile experiences on the interoceptive sensibility sub scales. The *ind* variables quantify the indirect effects, and *sum_ie* and *total* provide comprehensive measures of indirect and total effects, respectively.

3.1.1 Overall Model

In the 3.1.1 model, all mediators are included. All the other models derive from the 3.1.1 model but test only one mediator at a time. The syntax of the 3.1.1 model is reported below and it is called overall_med. Please note that *Eta_genitore* stands for maternal age.

```
overall_med <- '
f1_picts ~ a*TBQ_child + b1*MAIA_not_wor + b2*MAIA_attreg +
b3*MAIA_emoaw + b4*MAIA_selfreg + b5*MAIA_bodylis + b6*MAIA_trust + Eta_genitore
MAIA_not_wor ~ d1*TBQ_child
MAIA_attreg ~ d2*TBQ_child
MAIA_emoaw ~ d3*TBQ_child
MAIA_selfreg ~ d4*TBQ_child
```

```
MAIA_bodylis ~ d5*TBQ_child
MAIA_trust ~ d6*TBQ_child


# indirect and total effects
ind1 := b1*d1
ind2 := b2*d2
ind3 := b3*d3
ind4 := b4*d4
ind5 := b5*d5
ind6 := b6*d6


sum_ie := ind1 +ind2 +ind3 + ind4 + ind5 + ind6
total  := a + sum_ie
'
```

Model coefficients are resumed below.

```
Regressions:
             Estimate  Std.Err  z-value  P(>|z|) ci.lower ci.upper   Std.lv  Std.all
  f1_picts ~
   TBQ_child  (a)   0.260    0.063    4.151    0.000    0.137    0.383    0.260    0.233
   MAIA_nt_w (b1)  -0.008    0.051   -0.152    0.879   -0.108    0.092   -0.008   -0.008
   MAIA_ttrg (b2)   0.087    0.045    1.915    0.056   -0.002    0.176    0.087    0.098
   MAIA_emow (b3)   0.148    0.044    3.388    0.001    0.062    0.233    0.148    0.172
   MAIA_slfr (b4)   0.073    0.040    1.835    0.066   -0.005    0.152    0.073    0.094
   MAIA_bdyl (b5)   0.039    0.040    0.966    0.334   -0.040    0.117    0.039    0.050
   MAIA_trst (b6)  -0.057    0.039   -1.443    0.149   -0.134    0.020   -0.057   -0.077
   Eta_gentr        0.010    0.010    1.044    0.297   -0.009    0.029    0.010    0.053
```

```
  MAIA_not_wor ~
   TBQ_child (d1)   0.020   0.060   0.333   0.739  -0.097   0.137   0.020   0.018
  MAIA_attreg ~
   TBQ_child (d2)   0.182   0.067   2.696   0.007   0.050   0.314   0.182   0.144
  MAIA_emoaw ~
   TBQ_child (d3)   0.115   0.070   1.648   0.099  -0.022   0.253   0.115   0.089
  MAIA_selfreg ~
   TBQ_child (d4)   0.216   0.077   2.826   0.005   0.066   0.366   0.216   0.151
  MAIA_bodylis ~
   TBQ_child (d5)   0.257   0.076   3.360   0.001   0.107   0.406   0.257   0.179
  MAIA_trust ~
   TBQ_child (d6)   0.498   0.078   6.412   0.000   0.346   0.650   0.498   0.328

Intercepts:
             Estimate  Std.Err  z-value  P(>|z|) ci.lower ci.upper   Std.lv  Std.all
  .f1_picts      -2.374   0.526   -4.511   0.000   -3.406   -1.343   -2.374   -2.677
  .MAIA_not_wor    3.434   0.217   15.837   0.000    3.009    3.858    3.434    3.900
  .MAIA_attreg     2.986   0.244   12.235   0.000    2.508    3.465    2.986    2.982
  .MAIA_emoaw      4.032   0.254   15.901   0.000    3.535    4.529    4.032    3.901
  .MAIA_selfreg    2.733   0.277    9.859   0.000    2.190    3.277    2.733    2.401
  .MAIA_bodylis    2.412   0.277    8.723   0.000    1.870    2.954    2.412    2.114
  .MAIA_trust      2.382   0.281    8.473   0.000    1.831    2.933    2.382    1.972

Variances:
             Estimate  Std.Err  z-value  P(>|z|) ci.lower ci.upper   Std.lv  Std.all
  .f1_picts       0.691   0.053   13.077   0.000    0.587    0.794    0.691    0.878
  .MAIA_not_wor    0.775   0.059   13.077   0.000    0.659    0.891    0.775    1.000
```

```
  .MAIA_attreg     0.982   0.075  13.077   0.000   0.835   1.129   0.982   0.979
  .MAIA_emoaw      1.060   0.081  13.077   0.000   0.901   1.219   1.060   0.992
  .MAIA_selfreg    1.267   0.097  13.077   0.000   1.077   1.457   1.267   0.977
  .MAIA_bodylis    1.261   0.096  13.077   0.000   1.072   1.450   1.261   0.968
  .MAIA_trust      1.303   0.100  13.077   0.000   1.107   1.498   1.303   0.893

R-Square:
                   Estimate
    f1_picts          0.122
    MAIA_not_wor      0.000
    MAIA_attreg       0.021
    MAIA_emoaw        0.008
    MAIA_selfreg      0.023
    MAIA_bodylis      0.032
    MAIA_trust        0.107

Defined Parameters:
             Estimate  Std.Err  z-value  P(>|z|) ci.lower ci.upper   Std.lv  Std.all
   ind1        -0.000    0.001   -0.138    0.890   -0.002    0.002   -0.000   -0.000
   ind2         0.016    0.010    1.561    0.118   -0.004    0.036    0.016    0.014
   ind3         0.017    0.012    1.482    0.138   -0.006    0.040    0.017    0.015
   ind4         0.016    0.010    1.539    0.124   -0.004    0.036    0.016    0.014
   ind5         0.010    0.011    0.928    0.353   -0.011    0.031    0.010    0.009
   ind6        -0.028    0.020   -1.408    0.159   -0.068    0.011   -0.028   -0.025
   sum_ie       0.030    0.029    1.029    0.304   -0.027    0.088    0.030    0.027
   total        0.290    0.059    4.928    0.000    0.175    0.406    0.290    0.260
```

3.1.2 Not worrying model

The model 3.1.2 tests Not Worrying scale scores as mediator.

```
Regressions:
                   Estimate  Std.Err  z-value  P(>|z|) ci.lower ci.upper   Std.lv  Std.all
  f1_picts ~
    TBQ_child  (a)    0.286    0.060    4.779    0.000    0.169    0.403    0.286    0.252
    MAIA_nt_w (b1)    0.068    0.053    1.265    0.206   -0.037    0.172    0.068    0.066
    MAIA_ttrg         0.000                               0.000    0.000    0.000    0.000
    MAIA_emow         0.000                               0.000    0.000    0.000    0.000
    MAIA_slfr         0.000                               0.000    0.000    0.000    0.000
    MAIA_bdyl         0.000                               0.000    0.000    0.000    0.000
    MAIA_trst         0.000                               0.000    0.000    0.000    0.000
    Eta_gentr         0.006    0.010    0.637    0.524   -0.013    0.026    0.006    0.034
  MAIA_not_wor ~
    TBQ_child (d1)    0.020    0.060    0.333    0.739   -0.097    0.137    0.020    0.018

Intercepts:
                   Estimate  Std.Err  z-value  P(>|z|) ci.lower ci.upper   Std.lv  Std.all
   .f1_picts         -1.485    0.465   -3.195    0.001   -2.396   -0.574   -1.485   -1.650
   .MAIA_not_wor      3.434    0.217   15.837    0.000    3.009    3.858    3.434    3.900

Variances:
                   Estimate  Std.Err  z-value  P(>|z|) ci.lower ci.upper   Std.lv  Std.all
   .f1_picts          0.755    0.058   13.077    0.000    0.642    0.868    0.755    0.933
   .MAIA_not_wor      0.775    0.059   13.077    0.000    0.659    0.891    0.775    1.000
```

```
R-Square:
                   Estimate
    f1_picts          0.067
    MAIA_not_wor      0.000

Defined Parameters:
             Estimate  Std.Err  z-value  P(>|z|) ci.lower ci.upper   Std.lv  Std.all
   ind1          0.001    0.004    0.322    0.748   -0.007    0.010    0.001    0.001
   total         0.287    0.060    4.792    0.000    0.170    0.404    0.287    0.254
```

3.1.3 Attention regulation model

The model 3.1.3 tests Attention regulation scale scores as mediator.

```
Regressions:
             Estimate  Std.Err  z-value  P(>|z|) ci.lower ci.upper   Std.lv  Std.all
  f1_picts ~
   TBQ_child  (a)   0.251    0.059    4.255    0.000    0.136    0.367    0.251    0.222
   MAIA_nt_w        0.000                               0.000    0.000    0.000    0.000
   MAIA_ttrg (b1)   0.190    0.046    4.097    0.000    0.099    0.281    0.190    0.212
   MAIA_emow        0.000                               0.000    0.000    0.000    0.000
   MAIA_slfr        0.000                               0.000    0.000    0.000    0.000
   MAIA_bdyl        0.000                               0.000    0.000    0.000    0.000
   MAIA_trst        0.000                               0.000    0.000    0.000    0.000
   Eta_gentr        0.005    0.010    0.533    0.594   -0.014    0.025    0.005    0.028
  MAIA_attreg ~
   TBQ_child (d1)   0.182    0.067    2.696    0.007    0.050    0.314    0.182    0.144
```

```
Intercepts:
                   Estimate  Std.Err  z-value  P(>|z|) ci.lower ci.upper   Std.lv  Std.all
   .f1_picts         -1.778    0.440   -4.038    0.000   -2.641   -0.915   -1.778   -1.976
   .MAIA_attreg       2.986    0.244   12.235    0.000    2.508    3.465    2.986    2.982

Variances:
                   Estimate  Std.Err  z-value  P(>|z|) ci.lower ci.upper   Std.lv  Std.all
   .f1_picts          0.723    0.055   13.077    0.000    0.615    0.832    0.723    0.894
   .MAIA_attreg       0.982    0.075   13.077    0.000    0.835    1.129    0.982    0.979

R-Square:
                   Estimate
    f1_picts          0.106
    MAIA_attreg       0.021

Defined Parameters:
                   Estimate  Std.Err  z-value  P(>|z|) ci.lower ci.upper   Std.lv  Std.all
    ind1              0.035    0.015    2.252    0.024    0.004    0.065    0.035    0.031
    total             0.286    0.060    4.777    0.000    0.169    0.403    0.286    0.253
```

3.1.4 Emotional awareness model

The model 3.1.4 tests Emotional Awareness scale scores as mediator.

```
Regressions:
                   Estimate  Std.Err  z-value  P(>|z|) ci.lower ci.upper   Std.lv  Std.all
```

```
  f1_picts ~
   TBQ_child  (a)   0.266   0.058   4.578   0.000   0.152   0.380   0.266   0.235
    MAIA_nt_w        0.000                          0.000   0.000   0.000   0.000
    MAIA_ttrg        0.000                          0.000   0.000   0.000   0.000
   MAIA_emow (b1)   0.219   0.044   4.943   0.000   0.132   0.305   0.219   0.251
    MAIA_slfr        0.000                          0.000   0.000   0.000   0.000
    MAIA_bdyl        0.000                          0.000   0.000   0.000   0.000
    MAIA_trst        0.000                          0.000   0.000   0.000   0.000
   Eta_gentr        0.011   0.010   1.154   0.249  -0.008   0.031   0.011   0.059
  MAIA_emoaw ~
   TBQ_child (d1)   0.115   0.070   1.648   0.099  -0.022   0.253   0.115   0.089

Intercepts:
                   Estimate  Std.Err  z-value  P(>|z|) ci.lower ci.upper   Std.lv  Std.all
   .f1_picts         -2.312    0.451   -5.131    0.000   -3.195   -1.429   -2.312   -2.565
   .MAIA_emoaw        4.032    0.254   15.901    0.000    3.535    4.529    4.032    3.901

Variances:
                   Estimate  Std.Err  z-value  P(>|z|) ci.lower ci.upper   Std.lv  Std.all
   .f1_picts          0.709    0.054   13.077    0.000    0.603    0.815    0.709    0.873
   .MAIA_emoaw        1.060    0.081   13.077    0.000    0.901    1.219    1.060    0.992

R-Square:
                   Estimate
    f1_picts          0.127
    MAIA_emoaw        0.008
```

```
Defined Parameters:
                   Estimate  Std.Err  z-value  P(>|z|) ci.lower ci.upper   Std.lv  Std.all
    ind1              0.025    0.016    1.563    0.118   -0.006    0.057    0.025    0.022
    total             0.291    0.060    4.864    0.000    0.174    0.409    0.291    0.257
```

3.1.5 Self regulation model

The model 3.1.5 tests Self Regulation scale scores as mediator.

```
Regressions:
                   Estimate  Std.Err  z-value  P(>|z|) ci.lower ci.upper   Std.lv  Std.all
  f1_picts ~
    TBQ_child  (a)    0.249    0.059    4.211    0.000    0.133    0.365    0.249    0.220
    MAIA_nt_w         0.000                               0.000    0.000    0.000    0.000
    MAIA_ttrg         0.000                               0.000    0.000    0.000    0.000
    MAIA_emow         0.000                               0.000    0.000    0.000    0.000
    MAIA_slfr (b1)    0.172    0.041    4.212    0.000    0.092    0.252    0.172    0.217
    MAIA_bdyl         0.000                               0.000    0.000    0.000    0.000
    MAIA_trst         0.000                               0.000    0.000    0.000    0.000
    Eta_gentr         0.005    0.010    0.526    0.599   -0.014    0.025    0.005    0.027
  MAIA_selfreg ~
    TBQ_child (d1)    0.216    0.077    2.826    0.005    0.066    0.366    0.216    0.151

Intercepts:
                   Estimate  Std.Err  z-value  P(>|z|) ci.lower ci.upper   Std.lv  Std.all
   .f1_picts         -1.677    0.432   -3.882    0.000   -2.524   -0.830   -1.677   -1.864
   .MAIA_selfreg      2.733    0.277    9.859    0.000    2.190    3.277    2.733    2.401
```

```
Variances:
                   Estimate  Std.Err  z-value  P(>|z|) ci.lower ci.upper   Std.lv  Std.all
   .f1_picts          0.721    0.055   13.077    0.000    0.613    0.830    0.721    0.891
   .MAIA_selfreg      1.267    0.097   13.077    0.000    1.077    1.457    1.267    0.977

R-Square:
                   Estimate
    f1_picts          0.109
    MAIA_selfreg      0.023

Defined Parameters:
                   Estimate  Std.Err  z-value  P(>|z|) ci.lower ci.upper   Std.lv  Std.all
    ind1              0.037    0.016    2.347    0.019    0.006    0.068    0.037    0.033
    total             0.286    0.060    4.776    0.000    0.169    0.403    0.286    0.253
```

3.1.6 Body listening model

The model 3.1.6 tests Body Listening scale scores as mediator.

```
Regressions:
                   Estimate  Std.Err  z-value  P(>|z|) ci.lower ci.upper   Std.lv  Std.all
  f1_picts ~
    TBQ_child  (a)    0.244    0.059    4.107    0.000    0.128    0.361    0.244    0.216
    MAIA_nt_w         0.000                               0.000    0.000    0.000    0.000
    MAIA_ttrg         0.000                               0.000    0.000    0.000    0.000
    MAIA_emow         0.000                               0.000    0.000    0.000    0.000
```

```
    MAIA_slfr        0.000                              0.000    0.000    0.000    0.000
    MAIA_bdyl (b1)   0.167    0.041    4.089    0.000    0.087    0.248    0.167    0.212
    MAIA_trst        0.000                              0.000    0.000    0.000    0.000
    Eta_gentr        0.006    0.010    0.654    0.513   -0.013    0.026    0.006    0.034
  MAIA_bodylis ~
    TBQ_child (d1)   0.257    0.076    3.360    0.001    0.107    0.406    0.257    0.179

Intercepts:
                   Estimate  Std.Err  z-value  P(>|z|) ci.lower ci.upper   Std.lv  Std.all
   .f1_picts         -1.658    0.430   -3.860    0.000   -2.500   -0.816   -1.658   -1.843
   .MAIA_bodylis      2.412    0.277    8.723    0.000    1.870    2.954    2.412    2.114

Variances:
                   Estimate  Std.Err  z-value  P(>|z|) ci.lower ci.upper   Std.lv  Std.all
   .f1_picts          0.723    0.055   13.077    0.000    0.615    0.832    0.723    0.893
   .MAIA_bodylis      1.261    0.096   13.077    0.000    1.072    1.450    1.261    0.968

R-Square:
                   Estimate
    f1_picts          0.107
    MAIA_bodylis      0.032

Defined Parameters:
                   Estimate  Std.Err  z-value  P(>|z|) ci.lower ci.upper   Std.lv  Std.all
    ind1              0.043    0.017    2.596    0.009    0.011    0.075    0.043    0.038
    total             0.287    0.060    4.794    0.000    0.170    0.404    0.287    0.254
```

3.1.7 Trusting model

The model 3.1.7 tests Trusting scale scores as mediator.

```
Regressions:
                   Estimate  Std.Err  z-value  P(>|z|) ci.lower ci.upper   Std.lv  Std.all
  f1_picts ~
    TBQ_child  (a)    0.247    0.063    3.914    0.000    0.123    0.370    0.247    0.218
    MAIA_nt_w         0.000                               0.000    0.000    0.000    0.000
    MAIA_ttrg         0.000                               0.000    0.000    0.000    0.000
    MAIA_emow         0.000                               0.000    0.000    0.000    0.000
    MAIA_slfr         0.000                               0.000    0.000    0.000    0.000
    MAIA_bdyl         0.000                               0.000    0.000    0.000    0.000
    MAIA_trst (b1)    0.078    0.041    1.891    0.059   -0.003    0.158    0.078    0.104
    Eta_gentr         0.004    0.010    0.440    0.660   -0.015    0.024    0.004    0.023
  MAIA_trust ~
    TBQ_child (d1)    0.498    0.078    6.412    0.000    0.346    0.650    0.498    0.328


Intercepts:
                   Estimate  Std.Err  z-value  P(>|z|) ci.lower ci.upper   Std.lv  Std.all
   .f1_picts         -1.365    0.437   -3.123    0.002   -2.221   -0.508   -1.365   -1.517
   .MAIA_trust        2.382    0.281    8.473    0.000    1.831    2.933    2.382    1.972


Variances:
                   Estimate  Std.Err  z-value  P(>|z|) ci.lower ci.upper   Std.lv  Std.all
   .f1_picts          0.751    0.057   13.077    0.000    0.638    0.864    0.751    0.928
   .MAIA_trust        1.303    0.100   13.077    0.000    1.107    1.498    1.303    0.893
```

```
R-Square:
                   Estimate
    f1_picts          0.072
    MAIA_trust        0.107

Defined Parameters:
             Estimate  Std.Err  z-value  P(>|z|) ci.lower ci.upper   Std.lv  Std.all
   ind1          0.039    0.021    1.814    0.070   -0.003    0.080    0.039    0.034
   total         0.285    0.060    4.762    0.000    0.168    0.403    0.285    0.252
```

3.1.8 Null model

```
lavaan 0.6.15 ended normally after 18 iterations

  Estimator                                         ML
  Optimization method                           NLMINB
  Number of model parameters                        18

                                                  Used       Total
  Number of observations                           342         377

Model Test User Model:

  Test statistic                               814.270
  Degrees of freedom                                36
  P-value (Chi-square)                           0.000
```

```
Parameter Estimates:

  Standard errors                             Standard
  Information                                 Expected
  Information saturated (h1) model          Structured

Intercepts:
                   Estimate  Std.Err  z-value  P(>|z|) ci.lower ci.upper   Std.lv  Std.all
   f1_picts          -0.023    0.049   -0.482    0.630   -0.119    0.072   -0.023   -0.026
   MAIA_not_wor       3.504    0.048   73.606    0.000    3.411    3.597    3.504    3.980
   MAIA_attreg        3.628    0.054   67.002    0.000    3.522    3.734    3.628    3.623
   MAIA_emoaw         4.440    0.056   79.436    0.000    4.330    4.549    4.440    4.295
   MAIA_selfreg       3.498    0.062   56.808    0.000    3.377    3.618    3.498    3.072
   MAIA_bodylis       3.319    0.062   53.782    0.000    3.198    3.440    3.319    2.908
   MAIA_trust         4.140    0.065   63.386    0.000    4.012    4.268    4.140    3.428
   TBQ_child          3.534    0.043   82.182    0.000    3.449    3.618    3.534    4.444
   Eta_genitore      33.395    0.254  131.601    0.000   32.897   33.892   33.395    7.116

Variances:
                   Estimate  Std.Err  z-value  P(>|z|) ci.lower ci.upper   Std.lv  Std.all
   f1_picts           0.810    0.062   13.077    0.000    0.688    0.931    0.810    1.000
   MAIA_not_wor       0.775    0.059   13.077    0.000    0.659    0.891    0.775    1.000
   MAIA_attreg        1.003    0.077   13.077    0.000    0.853    1.153    1.003    1.000
   MAIA_emoaw         1.068    0.082   13.077    0.000    0.908    1.228    1.068    1.000
   MAIA_selfreg       1.297    0.099   13.077    0.000    1.102    1.491    1.297    1.000
   MAIA_bodylis       1.302    0.100   13.077    0.000    1.107    1.497    1.302    1.000
```

```
    MAIA_trust      1.459    0.112   13.077    0.000    1.240    1.678    1.459    1.000
    TBQ_child       0.632    0.048   13.077    0.000    0.538    0.727    0.632    1.000
    Eta_genitore   22.023    1.684   13.077    0.000   18.722   25.323   22.023    1.000
```

3.1.9 Main effect model

The model 3.1.9 tests the main effect model which includes only the predictor.

```
Regressions:
                   Estimate  Std.Err  z-value  P(>|z|) ci.lower ci.upper   Std.lv  Std.all
  f1_picts ~
    TBQ_child  (a)    0.286    0.060    4.779    0.000    0.169    0.404    0.286    0.253
    MAIA_nt_wr        0.000                               0.000    0.000    0.000    0.000
    MAIA_attrg        0.000                               0.000    0.000    0.000    0.000
    MAIA_emoaw        0.000                               0.000    0.000    0.000    0.000
    MAIA_slfrg        0.000                               0.000    0.000    0.000    0.000
    MAIA_bdyls        0.000                               0.000    0.000    0.000    0.000
    MAIA_trust        0.000                               0.000    0.000    0.000    0.000
    Eta_genitr        0.006    0.010    0.552    0.581   -0.014    0.026    0.006    0.029
  MAIA_not_wor ~
    TBQ_child         0.000                               0.000    0.000    0.000    0.000
  MAIA_attreg ~
    TBQ_child         0.000                               0.000    0.000    0.000    0.000
  MAIA_emoaw ~
    TBQ_child         0.000                               0.000    0.000    0.000    0.000
  MAIA_selfreg ~
    TBQ_child         0.000                               0.000    0.000    0.000    0.000
  MAIA_bodylis ~
```

```
    TBQ_child       0.000                               0.000    0.000    0.000    0.000
  MAIA_trust ~
    TBQ_child       0.000                               0.000    0.000    0.000    0.000

Intercepts:
                   Estimate  Std.Err  z-value  P(>|z|) ci.lower ci.upper   Std.lv  Std.all
   .f1_picts         -1.222    0.428   -2.855    0.004   -2.062   -0.383   -1.222   -1.359
   .MAIA_not_wor      3.504    0.048   73.606    0.000    3.411    3.597    3.504    3.980
   .MAIA_attreg       3.628    0.054   67.002    0.000    3.522    3.734    3.628    3.623
   .MAIA_emoaw        4.440    0.056   79.435    0.000    4.330    4.549    4.440    4.295
   .MAIA_selfreg      3.498    0.062   56.808    0.000    3.377    3.618    3.498    3.072
   .MAIA_bodylis      3.319    0.062   53.782    0.000    3.198    3.440    3.319    2.908
   .MAIA_trust        4.140    0.065   63.386    0.000    4.012    4.268    4.140    3.428

Variances:
                   Estimate  Std.Err  z-value  P(>|z|) ci.lower ci.upper   Std.lv  Std.all
   .f1_picts          0.759    0.058   13.077    0.000    0.645    0.873    0.759    0.937
   .MAIA_not_wor      0.775    0.059   13.077    0.000    0.659    0.891    0.775    1.000
   .MAIA_attreg       1.003    0.077   13.077    0.000    0.853    1.153    1.003    1.000
   .MAIA_emoaw        1.068    0.082   13.077    0.000    0.908    1.228    1.068    1.000
   .MAIA_selfreg      1.297    0.099   13.077    0.000    1.102    1.491    1.297    1.000
   .MAIA_bodylis      1.302    0.100   13.077    0.000    1.107    1.497    1.302    1.000
   .MAIA_trust        1.459    0.112   13.077    0.000    1.240    1.678    1.459    1.000

R-Square:
                   Estimate
    f1_picts          0.063
```

```
    MAIA_not_wor      0.000
    MAIA_attreg       0.000
    MAIA_emoaw        0.000
    MAIA_selfreg      0.000
    MAIA_bodylis      0.000
    MAIA_trust        0.000

Defined Parameters:
                   Estimate  Std.Err  z-value  P(>|z|) ci.lower ci.upper   Std.lv  Std.all
   dir                0.286    0.060    4.779    0.000    0.169    0.404    0.286    0.253
```

3.2 Final Comparison

```
                df      AIC
fit_med_notwor   8 1773.841
fit_med_attreg   8 1840.114
fit_med_emoaw    8 1859.340
fit_med_body     8 1925.618
fit_med_selfreg  8 1926.358
fit_med_trust    8 1949.575
fit             28 6900.348
fit_main        16 6976.289
fit0            18 9844.241
 model weights
[1] 1 0 0 0 0 0 0 0 0
```

Overall, as it clearly emerges from AIC values, mediation models outperformed the overall, the null and the main-effect models (Table 3.2). Considering the weights, AIC weights were lower than 0.001 for all the models except for the model with not-worrying scores as mediator where AIC weights were very close to 1.00. Thus, among all the tested mediators, the model with not-worrying scores resulted as the best one.

4 References

Akaike, H. (1974). A new look at the statistical model identification. IEEE transactions on automatic control, 19(6), 716-723.

Anderson, D. R., & Burnham, K. P. (2002). Avoiding pitfalls when using information-theoretic methods. The Journal of wildlife management, 912-918.

Bottesi, G., Ghisi, M., Altoè, G., Conforti, E., Melli, G., & Sica, C. (2015). The Italian version of the Depression Anxiety Stress Scales-21: Factor structure and psychometric properties on community and clinical samples. Comprehensive psychiatry, 60, 170-181.

Pitt, M. A., Myung, I. J., & Zhang, S. (2002). Toward a method of selecting among computational models of cognition. Psychological review, 109(3), 472.

Rosseel, Y., Oberski, D., Byrnes, J., Vanbrabant, L., Savalei, V., Merkle, E., ... & Jorgensen, T. (2017). Package 'lavaan'. Retrieved June, 17(1).

Vandekerckhove, J., Matzke, D., & Wagenmakers, E. J. (2015). Model comparison and the principle. The Oxford handbook of computational and mathematical psychology, 300.

Wagenmakers, EJ., Farrell, S. AIC model selection using Akaike weights. Psychonomic Bulletin & Review 11, 192–196 (2004). https://doi.org/10.3758/BF03206482
